# A Radiation-Pattern Reconfigurable Antenna Array for Vehicular Communications

**DOI:** 10.3390/s24134136

**Published:** 2024-06-26

**Authors:** Feng Gao, Hucheng Sun

**Affiliations:** Jiangsu Collaborative Innovation Center of Atmospheric Environment and Equipment Technology, Nanjing University of Information Science and Technology, Nanjing 210044, China; 202212490220@nuist.edu.cn

**Keywords:** pattern reconfigurable antenna, end-fire radiation, omnidirectional radiation, PIN diode

## Abstract

This paper presents a low-profile reconfigurable antenna array capable of five radiation-pattern modes for vehicular communication applications. The antenna array consists of four antenna elements, each containing four square patches. Exciting one of the square patches generates a broadside radiation. A square parasitic patch is added at the rear of the excited patch, and two square parasitic patches are placed at the front. By optimizing the design of these parasitic patches, including the treatment of center slotting and addition of shorting pins, the antenna element achieves an end-fire beam with a certain tilt angle. On this basis, a reconfigurable feeding network is designed with 1:1 and 1:4 output modes. By connecting the reconfigurable feeding network to the four antenna elements and altering the on/off states of the PIN diodes in the feeding network, a reconfigurable antenna with four end-fire beams and one omnidirectional beam in its radiation pattern is realized. Measurement results demonstrate an excellent impedance bandwidth, radiation pattern, and gain performance in all modes. The four end-fire and one omnidirectional radiation characteristics make it highly suitable for vehicular communication applications.

## 1. Introduction

With the rapid development of automobile intelligence and communication technology, the demand for more efficient and reliable communication solutions in vehicular communication systems is becoming increasingly urgent [1,2,3,4,5]. However, traditional fixed-pattern antennas are limited by their fixed radiation characteristics and cannot adapt to the changing communication needs of vehicles in different working environments. Therefore, reconfigurable antennas with radiation-pattern control have become a research hotspot to address this issue [6,7,8,9,10]. Reconfigurable antennas with radiation-pattern control can adjust their radiation direction by modifying their structure or feeding network, thereby adapting to different communication scenarios [11]. For example, during high-speed driving, it may be necessary to enhance the forward communication performance of the antenna, while in urban areas, a broader coverage may be required [12]. Therefore, the research on reconfigurable antennas aims to improve the performance and flexibility of vehicular communication systems [13]. Against this background, researchers are dedicated to developing reconfigurable antennas with radiation-pattern control technology to meet the demands of various vehicular communication application scenarios [14]. These studies aim to provide more flexible, efficient, and reliable communication solutions for the development of intelligent vehicles and vehicular communication systems, advancing vehicular communication technology to new heights.

Under normal circumstances, pattern reconfigurable antennas can be implemented in two ways. One method of control is to adjust the internal structure of the antenna through switches to achieve different radiation patterns [15,16,17,18,19,20]. For example, in reference [15], by changing the ON/OFF state of PIN diodes on the driven dipoles, a planar reconfigurable antenna with one omnidirectional beam and two directional beams is achieved. In each mode, the antenna achieved good impedance matching in the frequency range of 3.30 to 4.20 GHz, with a radiation efficiency of 84.0%. The other method of control is to connect the antenna to a switchable feeding network to produce adjustable radiation patterns [21,22,23,24,25,26]. For instance, in reference [21], an antenna consisting of four identical curved dipoles and a broadband reconfigurable feeding network is designed. By switching the ON/OFF state of PIN diodes in the reconfigurable feeding network, four different modes with directional radiation patterns in the azimuth plane are obtained, and the antenna’s impedance bandwidth reaches 33.6%. In comparison, when more radiation patterns are required, implementing the first method is more challenging. This is because the first method requires constructing different equivalent radiation structures within a fixed antenna structure, and ensuring good radiation characteristics under each state, which is more demanding. In contrast, in the second method, the radiation structure and the feeding network are separated. When constructing different equivalent feeding structures, the radiation characteristics of the antenna part will not be affected. Therefore, the reconfigurable feeding network can be freely designed to obtain more modes.

In vehicular communication, antennas need to have multiple radiation patterns to adapt to different communication needs and application scenarios [27]. Common radiation patterns include omnidirectional and directional radiations. Omnidirectional radiation pattern refers to the antenna exhibiting uniform radiation characteristics in the horizontal direction, with roughly equal radiation intensity along the horizontal plane [28]. This radiation pattern is suitable for broadcasting and coverage scenarios in vehicular communication, such as wireless network coverage provided by vehicle Wi-Fi hotspots. Directional radiation pattern refers to the antenna radiating higher power in a specific direction and lower power in other directions [29]. This radiation pattern is suitable for scenarios requiring signal transmission in specific directions, such as long-distance communication between vehicle communication systems and base stations. Considering the uncertainty of the relative orientation between the vehicle and its nearest base station while driving, it is preferable for the antenna to have coverage capability in all horizontal directions, requiring the antenna to achieve reconfigurable directional radiation beams in multiple directions [30]. Therefore, reconfigurable antennas used for vehicular communication need to have both omnidirectional and multiple directional radiation patterns.

In this work, a low-profile reconfigurable antenna array capable of providing five radiation modes is proposed for vehicular communication applications. The antenna array consists of four antenna elements capable of generating tilted directional beams. These four elements are arranged in a 90-degree rotation on the horizontal plane. In addition to designing the antenna elements, a reconfigurable feeding network with five different output modes is developed. By connecting the reconfigurable feeding network to the four antenna elements and changing the switching states in the feeding network, the antenna with five pattern modes reconfigurable is realized. This antenna possesses the capability to seamlessly transition between four directional beam modes and one omnidirectional beam mode, rendering it well-suited for vehicular communication applications.

## 2. Antenna Element Design

**Figure 1:**
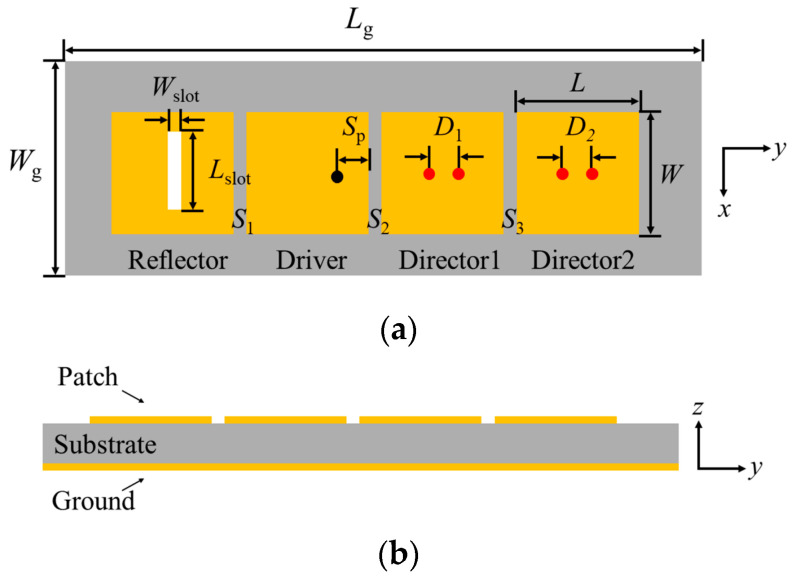
Layout of the antenna element: (**a**) Top view; (**b**) Side View.

Figure 1 illustrates the structure of the proposed microstrip patch antenna array element. The upper copper layer of this element is covered with four microstrip patches, while the lower copper layer serves as the ground plane, all printed on a 1.5 mm-thick F4B substrate with a dielectric constant of 4.5 and a loss tangent of 0.0015. Initially, one of the four patches is designated as the driver and fed using a coaxial probe, resulting in a broadside radiation. Then, a patch is added to the rear end of the driver patch, acting as a reflector. Typically, the electrical length of the patch reflector should be slightly greater than that of the driver patch. To increase the effective electrical length of the patch reflector, a narrow slot is etched onto the patch, interrupting the surface current path. This encourages the current to flow around the narrow slot, effectively increasing the path length of surface current flow and achieving the reflective effect. When the driver patch resonates at the center frequency, its impedance becomes purely resistive, and the addition of the patch reflector introduces inductive effects. Subsequently, two patches are added to the front end of the driver patch, serving as directors. At the center frequency, the director patches exhibit capacitive characteristics, thereby improving the impedance matching properties of the driver patch. Additionally, a pair of shorting pins is added to each of the director patches to further enhance their frequency characteristics of the antenna. By adding a patch reflector and two patch directors, and further optimizing the structure of each part, this antenna array element can generate an end-fire beam with a certain tilt angle. The software Ansoft HFSS 2020 was used to optimize the design of this antenna array element at a center frequency of 5 GHz. The optimized dimensional parameters of the antenna array element are listed in Table 1.

**Figure 2:**
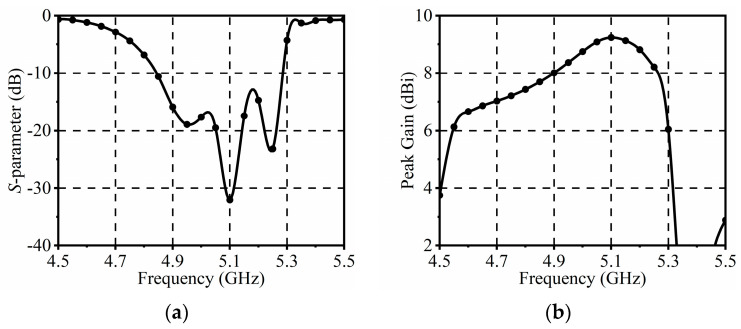
Simulated *S*-parameters and peak gains of the antenna element: (**a**) *S*-parameters; (**b**) Peak gains.

**Figure 3:**
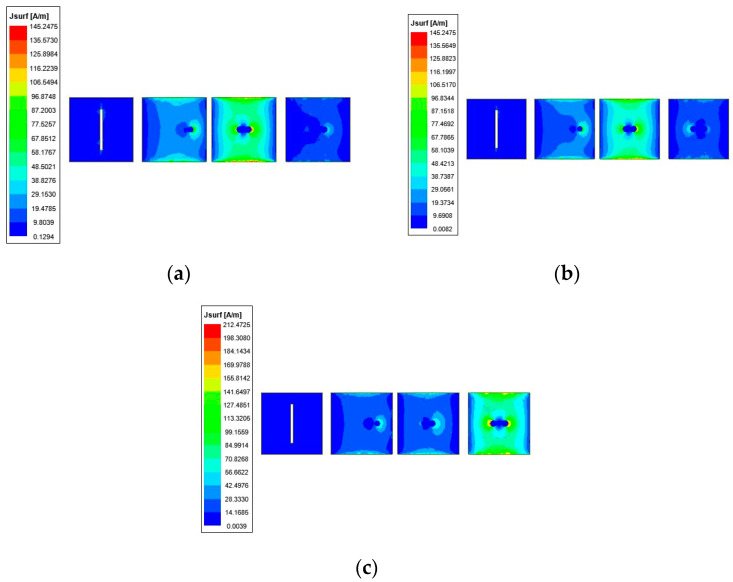
Simulated current distributions. (**a**) At 4.94 GHz. (**b**) At 5.1 GHz. (**c**) At 5.24 GHz.

Figure 2a shows the simulated *S*-parameters of the proposed antenna. It can be seen that the simulated −10 dB impedance bandwidth covers the frequency range from 4.84 to 5.28 GHz, which corresponds to a fractional bandwidth of 9%. Typically, microstrip patch antennas suffer from narrow bandwidths. In this design, the antenna’s bandwidth is extended to some extent by optimizing the reflector, driver, and directors. From Figure 2a, it can be found that three resonance points occur around the center frequency. As shown in Figure 3, the simulated current distributions at frequencies of 4.94 GHz, 5.1 GHz, and 5.24 GHz are provided. The first resonance at 4.94 GHz is generated by the capacitive self-impedance of the driver combined with the shunt inductance of director 1. At 5.1 GHz, the capacitive self-impedance of the driver decreases, allowing the shunt inductance of director 1 to dominate the second resonance. Furthermore, at 5.24 GHz, the shunt inductance of director 2 contributes to the third resonance. Optimizing the working frequencies corresponding to these structural parts can effectively broaden the bandwidth of the antenna. Figure 2b illustrates the peak gain of the antenna element, which varies from 7.2 to 9.2 dBi within the operating band. Also, the peak gain is relatively stable throughout the operating frequency range, with minimal impact on practical applications.

**Figure 4:**
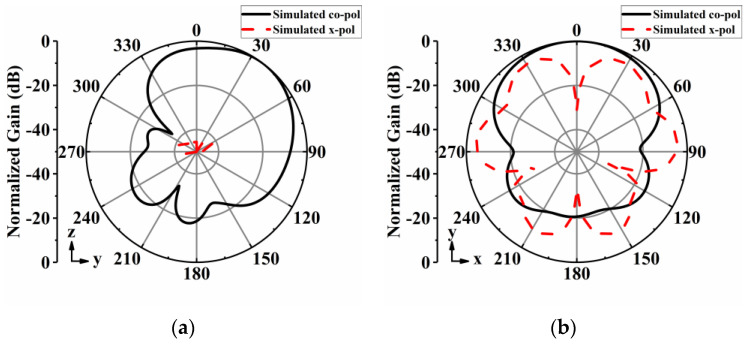
Simulated normalized radiation patterns of antenna element at 5 GHz: (**a**) *yoz*−plane; (**b**) *xoy*−plane.

Figure 4 shows the simulated normalized radiation patterns of the proposed antenna array element at 5 GHz. The maximum radiation direction of the antenna is deflected by approximately 37° toward the positive *y*-axis, with a half-power beamwidth of 67°. 

**Figure 5:**
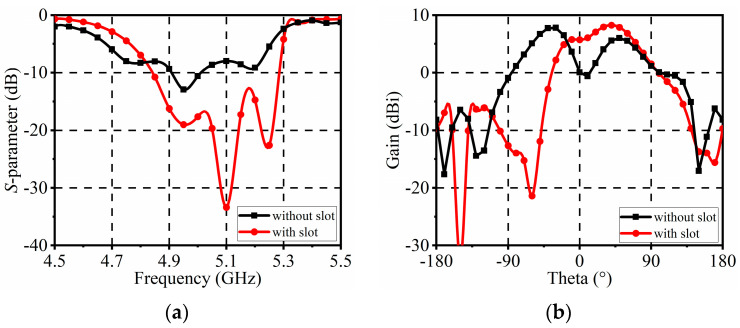
Simulated results of the antenna element with and without the narrow slot. (**a**) *S*-parameters; (**b**) Radiation patterns in the *yoz*−plane.

Figure 5 shows the simulated *S*-parameters and radiation patterns of the antenna elements with and without a narrow slot. As can be seen, with a narrow slot, the antenna element’s impedance matching is improved. Also, the main beam is deflected 40° from the +z-direction to the +y-direction. Therefore, the presence of the narrow slot turns the corresponding patch into a reflector, thereby improving impedance matching and deflecting the beam.

**Figure 6:**
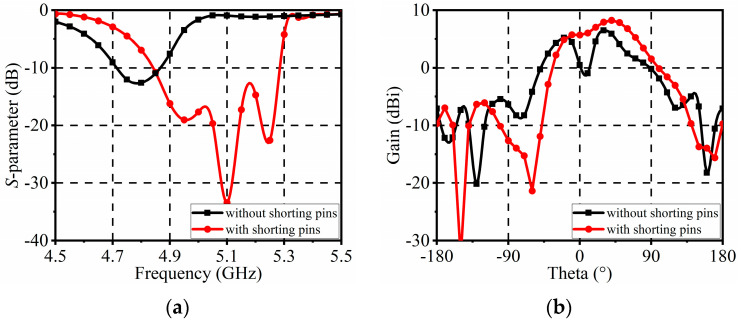
Simulated results of the antenna element with and without shorting pins. (**a**) *S*-parameters; (**b**) Radiation patterns in the *yoz*−plane.

Figure 6 shows the simulated S-parameters and radiation patterns of the antenna elements with and without shorting pins. As can be seen, after adding two pairs of shorting pins, the two patches act as directors. This improves the antenna’s impedance matching, expands its bandwidth, enhances the main beam, and increases the gain. Hence, the frequency characteristics are improved. The working principle involves the resonant condition of the patch driver, where its self-impedance becomes capacitive. The phase of the surface current density on this patch director lags behind that of the patch driver, thereby acquiring a directive function. Introducing two shorting pins creates an equivalent shunt inductance, which elevates the resonance frequency of this pin-loaded patch director. This increased resonance frequency, slightly above that of the patch driver, allows this parasitic element to effectively function as a patch director.

## 3. Pattern Reconfigurable Antenna Array

### 3.1. Antenna Configuration

**Figure 7:**
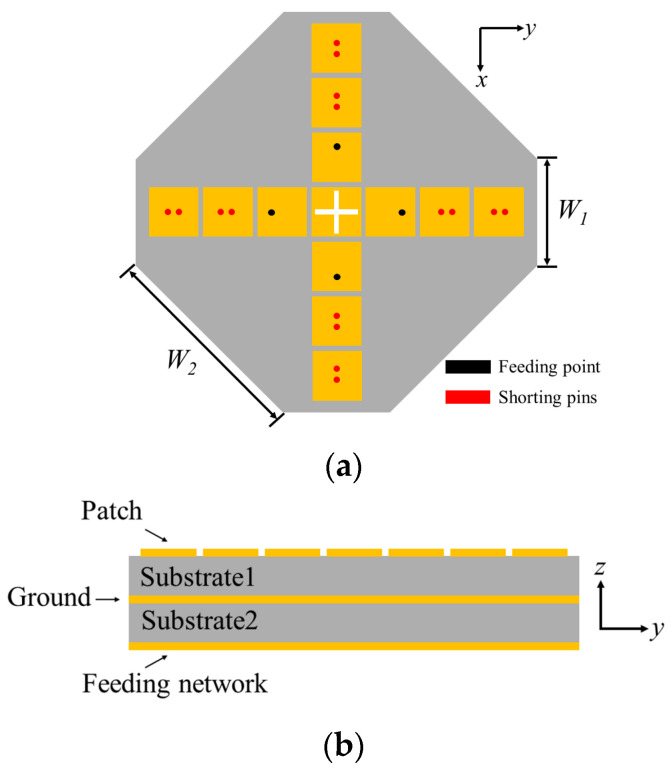
Layout of the array antenna: (**a**) Top view; (**b**) Side View.

The structure of the proposed pattern reconfigurable antenna array is shown in Figure 7. The antenna array mainly consists of four radiating antenna elements and a reconfigurable feeding network. Two layers of F4B substrates (*ε*_r_ = 4.5, tan *δ* = 0.0015) with thicknesses of 1.5 mm in the upper layer and 0.254 mm in the lower layer are utilized in the design. The radiating part of the antenna is printed on the upper copper layer, while the reconfigurable feeding network is placed on the lower copper layer.

Based on the optimized antenna array elements, an antenna array is formed by placing four identical antenna elements symmetrically in the ±x and ±y directions. These four groups of antenna elements share a common reflector, on which an orthogonal slot is etched to achieve a better radiation performance. All four patch drivers are fed by coaxial ports. To further increase the gain of the antenna array, one can use more directors or increase the thickness of the dielectric substrate. To suppress the omnidirectional gain fluctuation within 1 dB and simultaneously reduce the size of the antenna, the antenna shape is optimized to be an octagon. Then, by designing a reconfigurable feeding network and using it to feed the antenna array, the radiation pattern reconfigurable characteristics are achieved. Through controlling the switch states of the reconfigurable feeding network, the antenna array can freely switch between omnidirectional radiation mode and multiple directional radiation modes.

### 3.2. Reconfigurable Feeding Network

**Figure 8:**
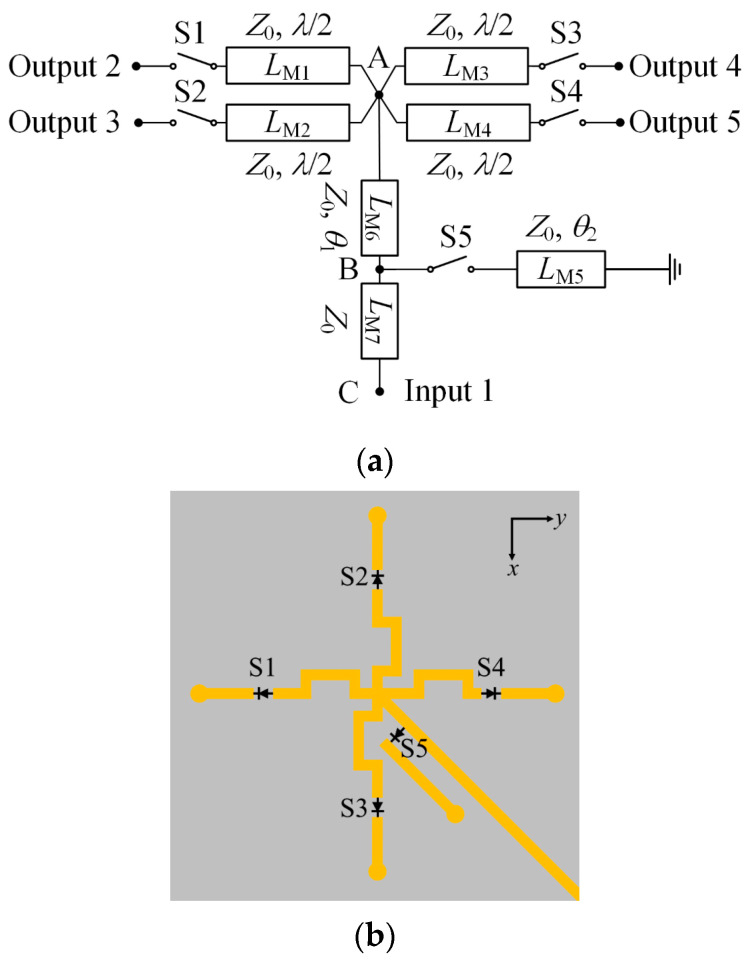
The proposed reconfigurable feeding network: (**a**) Schematic diagram; (**b**) Layout.

A reconfigurable feeding network with switchable impedance matching is proposed, as illustrated in Figure 8. The schematic diagram and layout of the network are provided. It comprises a single input port and four output ports, with each output port individually connected to four antenna elements. Within the feeding network, five switches, S1–S5, are employed to control its states. Microstrip lines *L*_M1_, *L*_M2_, *L*_M3_, and *L*_M4_ maintain a characteristic impedance of *Z*_0_ and an electrical length of half-wavelength. Hence, by altering the states of the switches S1–S4, lines *L*_M1_, *L*_M2_, *L*_M3_, and *L*_M4_ can be manipulated to connect with or disconnect from point A.

**Figure 9:**
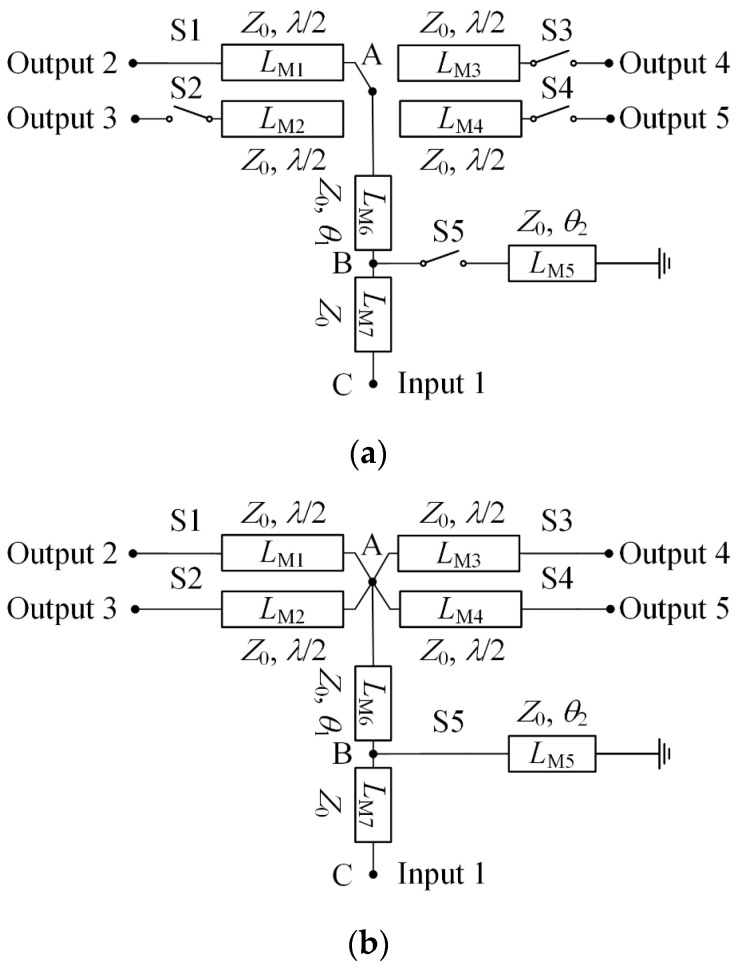
Equivalent circuit diagram: (**a**) 1:1 power mode; (**b**) 1:4 power mode.

The proposed reconfigurable feeding network enables the realization of four 1:1 modes and one 1:4 mode. In the four 1:1 power modes, the entire power is directed to only one output port. The equivalent circuit of the network in the first state (state I) of the four 1:1 modes is depicted in Figure 9a. When switches S2–S4 are turned off, lines *L*_M2_, *L*_M3_, and *L*_M4_ function as open circuits at node A. Additionally, with S5 turned off, line *L*_M5_ is disconnected from point B. As a result, the RF power input to port 1 can be completely routed to output port 2. Similarly, the remaining states (states II to IV) of the four 1:1 power modes can be achieved by turning off switches S1 and S5 while turning on one of the switches (S2–S4).

For the 1:4 power mode, the input power is evenly distributed among four output ports. The equivalent circuit for state V is shown in Figure 6b. In this state, switches S1–S4 are turned on, resulting in lines *L*_M1_, *L*_M2_, *L*_M3_, and *L*_M4_ being connected in parallel to node A. Consequently, the input impedance *Z*_A_ at node A becomes *Z*_0_/4. However, with line *L*_M6_, the input impedance *Z*_A_ is transformed to

(1) $$Z_{A}^{\prime }=Z_{0}\frac{Z_{A}+jZ_{0}\tan\theta_{1}}{Z_{0}+jZ_{A}\tan\theta_{1}}$$

which is mismatched with the feeding line *L*_M7_. To address this issue, switch S5 is turned on, and line *L*_M5_ severs as a matching stub. The input impedance of line *L*_M5_ is adjusted to

(2) $$Z_{5}=jZ_{0}\tan\theta_{2}$$

With the stub line *L*_M5_, the total input impedance *Z*_C_ at node C becomes

(3) $$Z_{C}=Z_{A}^{\prime }∥Z_{5}$$

By appropriately selecting the parameters *θ*_1_ and *θ*_2_, *Z*_C_ can be matched to the impedance of the feeding line. Following optimization, parameters *θ*_1_ and *θ*_2_ are determined to be 26.6° and 146.3° in the final design, respectively. Table 2 outlines the operational status of the switches for each power mode.

**Figure 10:**
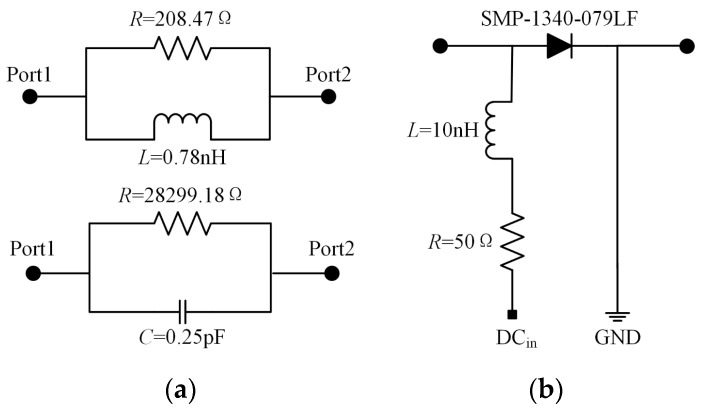
(**a**) Equivalent circuit models of the p-i-n diode at both ON and OFF states; (**b**) Equivalent circuit model of bias circuit.

The SMP1340-079LF PIN diodes have been chosen as the switches in the design of the feeding network. Due to the unavailability of manufacturer-provided data for these PIN diodes at 5 GHz, we conducted measurements illustrated in Figure 10a. Figure 7b depicts the diagram of the DC bias circuit for the PIN diodes, incorporating a 10 nH inductor and a 50 Ω resistor. Applying a DC bias voltage of 5 V results in a forward current through the PIN diode of 100 mA.

**Figure 11:**
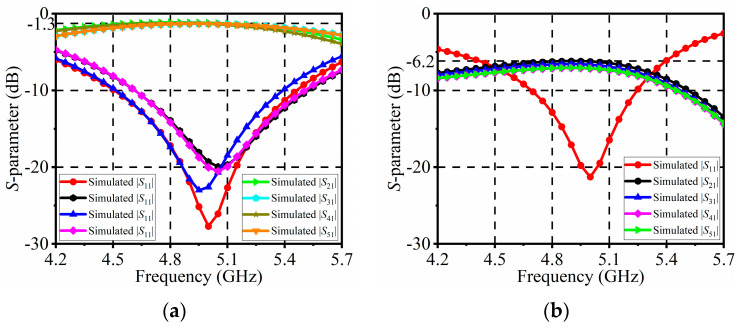
Simulated *S*-parameters of the reconfigurable feeding network: (**a**) 1:1 power modes; (**b**) 1:4 power mode.

The reconfigurable feeding network has undergone simulation and optimization. Figure 11 illustrates the simulated *S*-parameters across all power modes. In the four 1:1 power modes, the return loss at port 1 remains below −10 dB within the frequency range from 4.68 to 5.25 GHz, with corresponding insertion losses ranging from 1.22 to 1.35 dB. For the 1:4 power mode, the return loss at port 1 stays below −10 dB within the frequency range of 4.7 to 5.24 GHz, while the corresponding insertion losses are better than 7.16 dB.

### 3.3. Antenna Measurement and Evaluation

**Figure 12:**
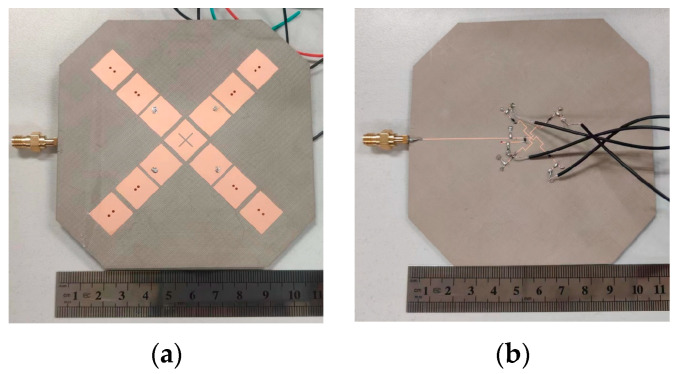
Photographs of the fabricated antenna array: (**a**) Top view; (**b**) Bottom view.

A radiation-pattern reconfigurable antenna array has been achieved by integrating the reconfigurable feeding network with four antenna elements. The antenna array has been fabricated and measured. Figure 12 presents the photographs of the fabricated antenna array.

**Figure 13:**
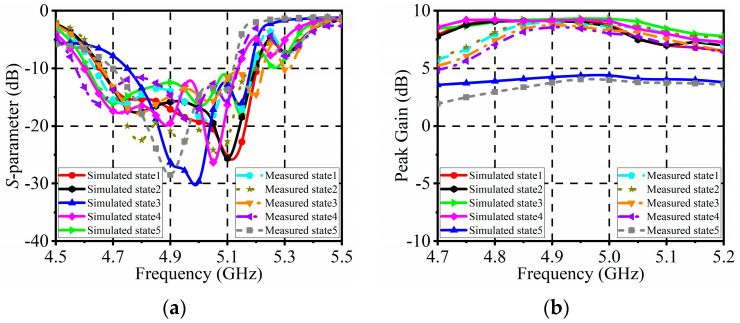
Simulated and measured results of the antenna array: (**a**) *S*-parameters; (**b**) Peak gains.

Figure 13 presents both the measured and simulated *S*-parameters and peak gains of the pattern reconfigurable antenna array across five operating states. Notably, the measured *S*-parameters exhibit a strong agreement with the simulated results across all five operating states. The −10 dB impedance bandwidth for each state spans from 4.65 to 5.2 GHz, covering an 11% frequency range. Regarding the peak gain, it surpasses 5 dBi for the four 1:1 power modes and 2.3 dBi for the 1:4 power mode. The performance of the antenna array can be influenced by various factors, including the soldering process between the antenna and the connectors, as well as the integration of PIN diodes onto the antenna. These factors may introduce discrepancies between the measurement results and the simulation results.

**Figure 14:**
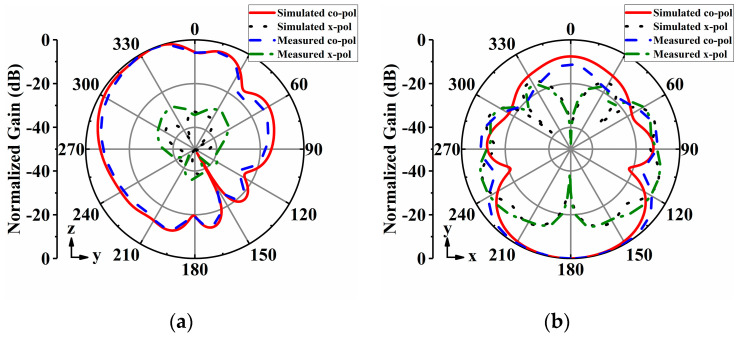
Simulated and measured radiation patterns of the array antenna for State I at 5 GHz: (**a**) *yoz*−plane; (**b**) *xoy*−plane.

**Figure 15:**
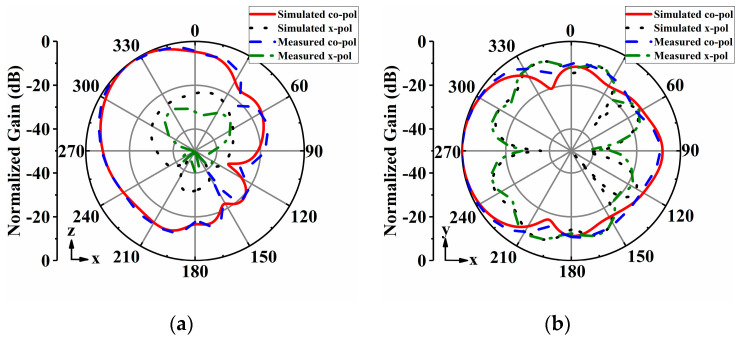
Simulated and measured radiation patterns of the array antenna for State II at 5 GHz: (**a**) *xoz*−plane; (**b**) *xoy*−plane.

**Figure 16:**
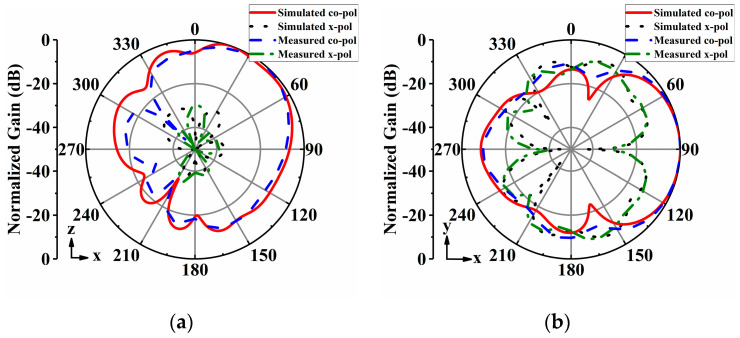
Simulated and measured radiation patterns of the array antenna for State III at 5 GHz: (**a**) *xoz*−plane; (**b**) *xoy*−plane.

**Figure 17:**
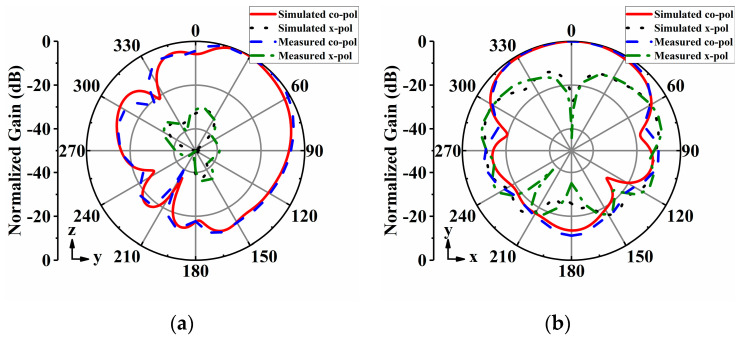
Simulated and measured radiation patterns of the array antenna for State IV at 5 GHz: (**a**) *yoz*−plane; (**b**) *xoy*−plane.

**Figure 18:**
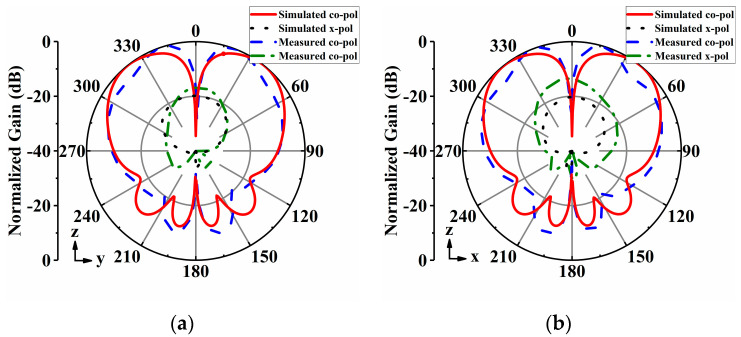
Simulated and measured radiation patterns of the array antenna for State V at 5 GHz: (**a**) *yoz*−plane; (**b**) *xoy*−plane.

The radiation patterns of the proposed antenna array were measured in an anechoic chamber. Figure 14, Figure 15, Figure 16, Figure 17 and Figure 18 display both simulated and measured radiation patterns at 5 GHz for all five operating states. Remarkably, the measured results agree well with the simulated ones across all operating states. It is worth noting that the measured cross-polarizations are slightly higher than the simulated ones. This deviation could primarily stem from fabrication tolerances and the influence of the DC bias circuits. In states I to IV, the antenna array exhibits directional radiation pointing toward ±x and ±y directions, making these radiation patterns suitable for scenarios requiring signal transmission in specific directions. Conversely, in state V, the antenna array displays omnidirectional radiation, which is ideal for broadcasting and coverage scenarios in vehicular communication.

Table 3 below presents the comparison between the reconfigurable antenna array in this work and the existing related works. It can be found that the proposed antenna array can switch between four directional radiation modes and one omnidirectional radiation mode. It is competitive with the existing arts in terms of profile, radiation modes, and radiation efficiency when used for vehicular communications applications.

## 4. Conclusions

A low-profile reconfigurable antenna array capable of providing five radiation modes tailored for vehicular communication applications has been proposed in this work. The array comprises four antenna elements generating tilted directional beams, arranged in a 90-degree rotation on the horizontal plane. Based on the antenna elements, a reconfigurable feeding network featuring five different output modes has been developed. By interconnecting the reconfigurable feeding network with the four antenna elements and manipulating the states of the switches within the feeding network, an antenna capable of reconfiguring into five distinct radiation patterns has been achieved. This antenna seamlessly transitions between four directional beam modes and one omnidirectional beam mode. With the advantages of a low profile and simple structure, it is highly suitable for various vehicular applications across different scenarios.

## Figures and Tables

**Table 1:** Dimensions of antenna elements.

Parameters	Value (mm)	Parameter	Value (mm)
*L*	13.4	*S*_3_	2
*W*	13.4	*S*_p_	3.3
*L*_slot_	8.5	*D*_1_	1.7
*W*_slot_	0.5	*D*_2_	2.5
*S*_1_	1.8	*L_g_*	100
*S*_2_	1.1	*W*_g_	60

**Table 2:** Switch Status for Each Power Mode.

Mode	S1	S2	S3	S4	S5	Radiation
1:1 (State I)	on	off	off	off	off	Directional
1:1 (State II)	off	on	off	off	off	Directional
1:1 (State III)	off	off	on	off	off	Directional
1:1 (State IV)	off	off	off	on	off	Directional
1:4 (State V)	on	on	on	on	on	Omnidirectional

**Table 3:** Comparison between the present design and the existing related arts.

Ref.	Size (*λ*_0_^3^)	Reconfigurability	No. of Diodes	Bandwidth (%)	Peak Gain (dBi)	Efficiency (%)
[31]	1.1 × 0.5 × 0.06	3-state beams	4	29	6.2	68
[32]	0.259 × 0.16 × 0.08	3-state beams	2	1.3	5.4	85
[21]	0.613 × 0.612 × 0.1	4-state beams	4	33.6	4.11	60
[33]	1.16 × 1.16 × 0.26	3-state beams	2	11.5	9.3	N.A.
[34]	0.57 × 0.57 × 0.04	4-state beams	2	8.2	2	N.A.
This work	2.4 × 2.4 × 0.03	5-state beams	5	11	9.3	88

## Data Availability

Data are contained within the article.
